# The Occurrence of Acute Pancreatitis in Adult Patients during a Measles Outbreak from November 2017 to May 2018 in Southeastern Serbia

**DOI:** 10.3390/medicina58111650

**Published:** 2022-11-15

**Authors:** Lidija Popović Dragonjić, Maja Jovanović, Miodrag Vrbić, Aleksandar Nastić, Miodrag Djordjević, Milica Veljković

**Affiliations:** 1 Department of Infectious Diseases and Epidemiology, Faculty of Medicine, University of Niš, 18000 Nis, Serbia; 2 Clinic for Infectology, University Clinical Center Niš, 18000 Nis, Serbia; 3 Department of Mathematics, Faculty of Sciences and Mathematics, University of Niš, 18000 Nis, Serbia; 4 Department Physiology, Faculty of Medicine, University of Niš, 18000 Nis, Serbia

**Keywords:** measles, acute morbillous pancreatitis, complications, amylase

## Abstract

*Background and Objectives:* Although it is believed that measles infections are under control, there is a global reappearance, and their treatment has become more complex as the disease is followed by a relatively high incidence of complications. This study, conducted on patients during a measles outbreak from November 2017 to May 2018, aims to evaluate a rarely reported complication of measles, acute morbilous pancreatitis (AMP), which has been reported in several cases to date. *Materials and Methods:* A total of 207 patients admitted and treated at the Clinic for Infectious Diseases, Clinical Center Nis, for measles infection were included in the analysis. The data collected from the patient’s medical records included the demographic characteristics, disease duration, full blood, serum, and urine biochemical analysis, general measles-associated symptoms, and disease outcome. *Results:* According to the serum and urine amylase activity, and some clinical symptoms AMP were diagnosed in 14% (29/207) of the studied patients. These patients had significantly higher levels of ALT and vomited more frequently than the patients without AMP. Only slight differences in measles duration, changes in RBC count, and CRP levels were found between the males and females with AMP. *Conclusions:* Acute morbillous pancreatitis should not be underestimated as a complication, even though according to the results of our survey, it was not associated with a fatal outcome or disease severity as the course of it can be frequently rapid and fatal.

## 1. Introduction

Measles has been re-emerging worldwide and the number of cases is increasing in all age groups, including adults. The infection is caused by an RNA virus from the Paramyxoviridae family, which is known to be quite contagious, as from a single infected person, 12–18 secondary infections occur [1]. Some time ago, it was believed that the virus could be eliminated; however, due to changes in human lifestyles, as well as in attitudes towards the immunization process, there has been an increased occurrence of cases of measles and sporadic outbreaks worldwide [2]. The last such outbreak that was noted on the territory of the Republic of Serbia was in 2017 and 2018, with an incidence rate of 9.8/100,000 and 70.3/100,000, respectively [3,4]. In the Southeastern Serbia region in 2018, the specific incidence rate at the age of 35–39 years was 223/100,000.

Possible complications caused by measles range from mild symptoms to multiorgan involvement, which sometimes results in fatal outcomes [5]. Complications often include otitis media, laryngotracheobronchitis, pneumonia, stomatitis, and diarrhea. Acute disseminated encephalomyelitis and sub-acute sclerosing panencephalitis are rare neurological complications, but their occurrence should not be overlooked [6]. Measles affects the gastrointestinal system in the largest number of patients, ranging from mild symptoms (e.g., nausea) to some serious life-threatening conditions. Typically, measles is detected as ‘‘giant cells’’ in the stomach and appendix tissue [7]. The most frequent complication of the gastrointestinal tract associated with measles is diarrhea, but some of the other frequent ones include acute appendicitis with perforation and peritonitis, mesenteric lymphadenitis, hepatitis, and ileocolitis [6,8]. Interestingly, acute morbillous pancreatitis (AMP) is the least known gastrointestinal complication [8], and up until now, only several cases of AMP have been yet reported in the literature [8,9,10,11,12].

As the natural course of measles and its complications in adults has not been well understood, the primary goal of this study was to present and elaborate on the clinical and bio-humoral characteristics of the adult patient population with AMP hospitalized at the Clinic for Infectious Diseases, Nis, Serbia, during the measles outbreak, in order to potentially understand the unexpectedly frequent occurrence of acute pancreatitis.

## 2. Materials and Methods

### 2.1. Study Design and Data Collection

The measles outbreak took place in the Nisava and Toplica districts in Southeastern Serbia for the period from 20 November 2017 until 8 May 2018. There were 1473 outpatients examined for measles by a specialist at the Clinic for Infectious Diseases, Nis, Serbia, out of which measles were confirmed in 1240 patients. The study protocol was presented and approved by the Medical Ethical Committees of University Clinical center Nis in Serbia (decision number: 28355/13) and the Faculty of Medicine University of Nis (Nis, Serbia). Informed consent was obtained from all of the patients before the commencement of the study. All of the patients’ data during the hospitalization and preparation of this database were double checked by three infectiology specialists (L.P.D., M.J., and M.V.) in order to secure a non-biased approach to patient selection and diagnosis.

The diagnosis of measles was established based on the following WHO criteria:

(i) Clinical manifestations (fever, cough, conjunctivitis, coryza, characteristic Koplik spots, or the appearance of a maculopapular rash).
(ii) Serological analysis (positive measles-specific immunoglobulin M (IgM)).

Out of a total of 224 hospitalized adult patients, 207 adult patients were further analyzed and processed, 130 females and 77 males, as these patients’ complete data from their medical histories were available. The data collected from the patient’s medical records were as follows: (i) demographic characteristics (age and gender); (ii) disease duration; (iii) full blood, serum, and urine biochemical analysis; (iv) general measles-associated symptoms; and (v) disease outcome.

For the diagnosis of AMP to be met, we followed the guidelines, where the first two criteria had to be fulfilled, and the third being optional [13,14]:

(i) Serum amylase activity is at least three times greater than the upper limit of normal (above 300 IU/L).
(ii) Urinary amylase activity above 2000 IU/L.
(iii) Abdominal pain suggestive of pancreatitis.

The connection between the serum CRP values and urine amylase was considered using a stratified sample of subjects with AMP where the CRP serum values were increased by less than 2-fold (category 1), between 2- and 3-fold (category 2), between 3- and 4-fold (category 3), and equal or larger than 4-fold (category 4). As the association between CRP values and AMP in patients with measles was examined in this study, at the commencement of this study we included only the patients without bacterial coinfection. The absence of bacterial infection was confirmed either through the absence of any clinical manifestations and/or negative culture of either nasal or throat swabs or sputum or urine sample. In addition, only patients with diarrhea without any detectable pathological microbiota were taken into consideration for the analysis.

### 2.2. Statistical Analysis

Data are presented as frequency distributions expressed as percentages or median/mean values. Normality distribution of continuous variables was confirmed by the Kolmogorov–Smirnov test. The comparison of mean values was done by either Student’s *t*-test or Mann–Whitney U test, depending on the variables’ distribution. Categorical variables were compared using a Chi-squared or Fischer’s exact test, depending on group size. Patients with missing data were excluded from the study at its very beginning when the data were collected. The statistical package SPSS (version 21.0, Armonk, NY, USA: IBM Corp, 2012) was used for all of the statistical data processing [15]. A two-sided *p* < 0.05 was considered to be statistically significant. 

## 3. Results

### 3.1. Characteristics of Patients with Acute Morbilous Pancreatitis

Around 0.26% of population had been measles patients, which was calculated based on the total number of inhabitants in the two studied regions (Nisava and Toplica, with around 460,000 inhabitants) obtained from the official population lists. A total of 207 adult patients, 130 female (63%) and 77 male (37%), with a mean age of 36 ± 10 years who were hospitalized during the measles epidemics outbreak in 2017/2018 at the Clinic for Infectious diseases, Clinical Center Nis, were included in this study. All of the patients presented with an increased body temperature (>38.2 °C) and generalized rash, while Koplik spots were observed in only 31.1 % of patients. The most common complication was pneumonia (43%), followed by hepatic involvement with various degrees of increase in the activity of liver enzymes and dyspeptic symptoms (43%). One patient developed acute respiratory distress syndrome (ARDS). There were two lethal outcomes, one patient with ARDS and one with non-ARDS.

Out of all of the hospitalized patients with measles, 29 (14%) were diagnosed with AMP as well. Among them, 14 were male and 15 were female, which made the distribution between genders almost equal (>0.05, Table 1). The age of patients with AMP was found to be almost the same as that of patients without AMP (Table 1). Regarding the total duration of disease (measle symptom duration) as well as the duration of hospitalization, it was found that they did not differ among the studied patients and were around 11 and 7 days, respectively (Table 1). None of the patients with AMP died, while as previously stated, two patients infected with measles, but without AMP, died.

The comparison of the blood cells data; WBC, RBC, and PLT count; neutrophile percent abundance; and Hgb between the patients with or without AMP revealed that the RBC count and Hgb concentration were statistically significantly higher in patients with AMP than in the group of patients without AMP (Table 1). Other values of the studied blood cells were more or less equal in the two studied groups.

The comparisons of a panel of serum biochemical analyses from patients with and without AMP are given in Table 1. The serum activity of ALT, γ-GT, and amylase was found to be significantly increased in patients with AMP, while the activity of LDH and the direct bilirubin concentrations were significantly decreased in the same group of patients (Table 1). Furthermore, the activity of amylase in the urine of patients with AMP was statistically significantly increased compared with the values measured in the patients without AMP (Table 1).

Out of the four traced measles-associated gastrointestinal clinical signs (nausea, vomiting, diarrhea, and abdominal pain), the statistical analysis revealed that vomiting was statistically significantly more frequent in patients with AMP (Table 1), while the remaining three signs were almost identical in both groups. Vomiting was present in around 92% of total patients who had AMP, and in around 53% of those without AMP. Other traced clinical signs, although they occurred slightly often in the group of patients with AMP, did not reach a significance level (Table 1).

### 3.2. Gender Differences in the Characteristics of Patients with Acute Morbilous Pancreatitis

When comparing the age of patients with AMP by their gender, no statistically significant differences were noted (Table 2). The disease duration was found to be statistically significantly longer in females than in males (*p* = 0.038), with a mean value of around 13 days, while the hospitalization time was almost identical in both genders and was between 7 and 8 days (Table 2).

The values of the serum parameters associated with liver tissue damage, (ALT, total and direct bilirubin) were found to be statistically significantly increased in male patients suffering from AMP compared with the female patients. Furthermore, CRP values pre-hospitalization and during hospitalization were found to be significantly higher in females than in males with AMP (Table 2). The serum amylase activity was found to be statistically significantly higher in female patients with AMP, while urinary amylase activity was detected to be statistically significantly higher in male patients with AMP (Table 2).

A comparison of the studied clinical parameters associated with AMP in patients of different genders revealed that they did not mutually differ (Table 2). Some symptoms such as nausea and vomiting were almost identically occurring in both genders, while reports of diarrhea and abdominal pain were less frequently found in males than in females (Table 2).

### 3.3. Association between AMP and CRP Changes

Changes in the CRP concentrations prior to and during hospitalization were distributed in four categories. Afterwards, only the patients without any detectable additional infection were included in the comparison between the group of patients with and without AMP (Table 2). No significant differences were noted between the changes in the CRP values in patients with and without AMP (Table 3). However, it should be noted that the majority of patients with AMP had a 2–3 times increase in CRP values (category 2), while in the group of patients without AMP, the CRP value increase was spread through the categories unevenly.

## 4. Discussion

Prior to the COVID pandemic during 2017/2018, there was a relatively large outbreak of measles in the Republic of Serbia, reaching an incidence rate of around 71 patients per 100,000 people [3,4]. The studied regions of the Republic of Serbia included in this study, Nišava District and Toplica District, accounted for nearly 25% of all patients during this outbreak. The hospitalized patients’ characteristics (Table 1) were similar in terms of the mean age, gender, clinical signs, and laboratory results when compared with the same data presented in recent studies that included adult patients with measles complications [16,17].

Complications arriving from measles infections have been reported in every organ system. Many of them are caused by a disruption of epithelial surface and immunosuppression, thus the respiratory and gastrointestinal complications in measles are the most frequent ones [7]. However, gastrointestinal complications are the most frequent ones, which can be judged by the symptoms from the gastrointestinal tract such as nausea and vomiting, which were present in more than half of the studied patients here (Table 1). Other complications include diarrhea, hepatitis, appendicitis with perforation and peritonitis, mesenteric lymphadenitis, and ileocolitis, as described in other studies [7,8]. A serious course of disease and complications associated with measles is certainly the cause of hospitalization and prolonged disease duration (Table 1). Interestingly, AMP was not found to affect the duration of the disease or the hospitalization time (Table 1); however, the duration of diseases in female patients with AMP was found to be significantly longer than the one in males suffering from AMP (Table 2). Similar data were obtained from a larger study of acute pancreatitis (AP) where females were the ones with prolonged hospitalization periods, thus indirectly having a longer disease duration [18].

Acute morbillous pancreatitis is rarely described in the literature, with our survey conducted on papers back to 1996 using Scopus and Google Scholar with keywords ‘’measles, pancreatitis’’, and only several case reports having been presented up until now [8,9,10,11,12]. In our study out of 207 hospitalized patients with measles, AMP, diagnosed according to the criteria for pancreatitis, was present in 14% (Table 1). The patients described as having AMP did not differ from the pancreatitis of other etiologies [8,9,10,11,12]. Recent progress in imaging studies and laboratory examinations of pancreatic enzymes has resulted in relatively precise criteria for acute pancreatitis [19]. The revised clinical criteria claim that AP is developed when at least two of the following conditions are present: (i) acute pain and tenderness in the upper abdomen; (ii) elevated pancreatic enzyme levels in the blood and/or urine; and (iii) ultrasound (US), CT, or magnetic resonance imaging (MRI) abnormalities of the pancreatic tissue characteristic for AP. However, the serum amylase level declines fast within 3 days after the onset of AP, while increased values for serum lipase persist for a longer period. The revised diagnostic criteria prefer the pancreatitis-specific enzymes in the serum and/or urine, such as pancreatic-type amylase and lipase [18] over other methods. 

In the present study, epidemiological and technical circumstances determined the diagnosis of acute pancreatitis. Given that, for epidemiological reasons, we could not perform imaging techniques located at a distant department, and that the biochemical laboratory lacked lipase reagents, AMP diagnostics was mainly based on the pancreatic-type amylase in the serum and urine. Initial symptoms that led us to suspect AMP were abdominal pain and dyspepsia, and, as stated in the inclusion criteria, only patients with either a triple increase in serum amylase activity or a urinary amylase activity above 2000 IU/L, which was in accordance with the findings of other studies [13,20]. Thus, in order to increase the accuracy for establishing the AMP, we used the highest suggested cut-off values of amylase suggested in the mentioned studies. In addition, the additional confirmation and avoidance of a false diagnosis of AMP was the fact that urine amylase, used as a criterion, may remain elevated for up to 2 weeks after the acute episode of pancreatitis [13,20]. The final confirmation of these criteria comes from the fact that statistically significant differences in the serum and urine amylase activity were found between the patients with and without AMP (Table 1). The severity of AMP, according to the values of amylase serum, was found to be more pronounced in females, while if we estimated the severity of AMP based on the values of the urinary amylase, the disease would be more pronounced in the male gender (Table 2). Generally, it is hard to conclude the severity of the AMP according to gender as all of the patients recovered and were discarded from the hospital within a two week period (Table 1 and Table 2).

Although abdominal pain is the main symptom of AP, in the case of our patients, vomiting was more common in patients with AMP than in those without, while abdominal pain was present without statistical significance in both groups. The reason probably lies in the systemic character of measles, because viral replication takes place in the gastric and intestinal mucosa, as well as in the cells of the conjunctiva and lungs [21]. Interestingly, none of the traced clinical symptoms were found to be different when they were compared in AMP patients of different genders (Table 2). Thus, the clinical symptoms should not be the sole criteria for AMP, as they might be misleading or overlap with the ones of another gastrointestinal disorder.

The obtained data suggest that some patients, apart from those with AMP, might have had some other complications, especially liver complications. These conclusions could be drawn from the fact that ALT, a liver-specific enzyme, and γ-GT are statistically significantly increased in patients with AMP (Table 1). The more pronounced hepatic damage was further found in male patients with AMP, where the ALT activity and bilirubin levels were significantly higher than in the females (Table 2) in the current study, although no gender differences were found in the severity of AP according to another study [18]. Liver failure is a common complication of pancreatitis, where around 85% of patients develop this disorder [22]. The pathophysiological mechanisms are various and on the one hand depend on the pancreatitis etiology, and on the other hand on the activity of proteolytic enzymes secreted by the exocrine pancreas and on the balance between proinflammatory and anti-inflammatory cytokines [22].

The absence of a relationship between an increase in CRP value (as well as the intensity of its increase during 3 days) and the appearance of AMP supports the fact that it was not a case of serious forms of AP. This can be concluded based on the fact that CRP values and the change in its serum values were useful for the assessment and monitoring of AP [23]. The intensity of the inflammatory reaction, both prior to and during hospitalization, was found to be higher in females with AMP (Table 2). The generally higher values of CRP in females [24] could explain the relatively higher values of this protein during a reaction to morbilli infection rather than to AMP per se.

Although bile stones and alcoholism are the most common cause of AP, approximately 10% of all cases are caused by different microorganisms. Most commonly, these include viruses (mumps, Coxsackie B, CMV, and hepatitis), bacteria (Mycoplasma pneumoniae and Leptospira), and parasites (Ascaris lumbricoides, Fasciola hepatitis, and Echinococcus). Each microorganism causes AP via different pathophysiological mechanisms [25]. 

The possible explanation of why measles causes AP could be explained through the receptor theory. There are two cell surface receptors for the measles virus, CD46 and signaling lymphocyte-activation molecule (SLAM)/nectin 4/CD150. The entrance into cells is mediated by glycoproteins of the virus envelope, hemagglutinin (H), which mediates binding, and fusion protein (F), which leads to membrane fusion [26,27]. A strong expression of the CD46 receptor was observed in the epithelial cells of the excretory ducts and exocrine glands, such as the salivary, pancreatic, renal tubules, and glomerular epithelium [28]. Thus, this is a reasonable explanation of how this virus provokes AP in infected persons. Another explanation could be the genotype-related measles virus affinity for specific tissues such as pancreatic tissue. Only two of the 24 known measles virus genotypes are responsible for outbreaks in Europe, including Serbia, starting in 2016: B3 and D8, according to the Measles Nucleotide Surveillance (MeaNS) database [29]. These genotypes mainly differ in the H protein, which again points to the potential direct interaction in the pancreatic tissue at the cellular level.

This study has a few limitations. The first limitation is the lack of a lipase reagent, which would help clarify the AMP diagnosis, without the need to analyze other enzymes and interpret those results to establish a diagnosis. Another shortcoming is a lack of abdominal ultrasonography in patients, which was not plausible here due to epidemiological reasons.

## 5. Conclusions

The relatively high proportion of AMP in hospitalized patients with measles in our survey (29/207) emphasizes that this complication should not be underestimated, even though presently it was not associated with a fatal outcome or severity. Its importance is not only in the fact that it is presented with uncomfortable gastrointestinal symptoms, but also in possible pancreatic complications of a delayed type and the necessity for patient follow-up. One thing is clear, when treating a patient with measles, one should not overlook the possibility of an AMP in these patients. The treatment of AMP, in a form of a supportive and rehydration therapy, might not come as a challenge when recognized and managed on time; however, pancreatitis does tend to have serious/fatal outcomes that develop quickly. The results of the present study are of great importance as there is a largely unknown area of the measles viral pathophysiology, infection diagnosis, and disease management, as well as the basic immunological aspects of measles infection.

## Figures and Tables

**Table 1:** Descriptive data and statistical analysis obtained from subjects with and without acute morbillous pancreatitis.

	Acute Morbillous Pancreatitis		*p*-Value
	Yes (*n* = 29)	No (*n* = 178)	
*Demographic characteristics*			
Mean age ± SD	35.5 ± 9.7	36.3 ± 10.8	>0.05
Gender (*n* (%))			
Male	14 (6.8)	63 (30.4)	>0.05
Female	15 (7.2)	115 (55.6)	
*Disease duration (mean values ± SD)*			
Total disease duration (days)	11.3 ± 3.8	12.8 ± 4.3	>0.05
Hospitalization time (days)	7.7 ± 3.5	6.9 ± 2.4	>0.05
*Blood cell analyses (mean values ± SD)*			
Maximum WBC count (× 10^5^)	5.9 ± 2.5	6.2 ± 3.4	>0.05
Neutrophile percent abundance	78.2 ± 11.2	75.9 ± 15.4	>0.05
RBC count (× 10^12^)	4.7 ± 0.1	4.4 ± 0.5	*0.0053*
Hgb (g/L)	142.9 ± 4.2	131.1 ± 14.7	*0.003*
Platelets count (× 10^6^)	179 ± 97	164 ± 78	>0.05
*Serum biochemical analyses (mean values ± SD)*			
LDH (U/L)	853 ± 132	977 ± 155	*<0.001*
AST (U/L)	110 ± 93	101 ± 58	>0.05
ALT (U/L)	162 ± 17.4	119 ± 13.6	*<0.001*
γ-GT (U/L)	242 ± 25	191 ± 45	*<0.001*
Total bilirubin (μmol/L)	12 ± 5.1	14.8 ± 11.2	>0.05
Direct bilirubin (μmol/L)	4.6 ± 3.5	10.1 ± 4	*<0.001*
CRP before hospitalization (mg/L)	36.5 ± 22.2	31.4 ± 21.5	>0.05
CRP during hospitalization (mg/L)	55.1 ± 29	62.5 ± 30	>0.05
Amylase (U/L)	423 ± 73	219 ± 103	*<0.001*
*Urine biochemical analyses (mean values ± SD)*			
Amylase (U/L)	3320 ± 852	811 ± 357	*<0.001*
*General measles-associated symptoms (number of cases (%))*			
Nausea (*n* (%))	24 (85.7)	112 (71.8)	>0.05
Vomiting (*n* (%))	22 (91.5)	83 (52.2)	*<0.001*
Diarrhea (*n* (%))	12 (50)	49 (33)	>0.05
Abdominal pain (*n* (%))	9 (50)	35 (33)	>0.05

WBC—white blood cells; RBC—red blood cells; Hgb—hemoglobin.

**Table 2:** Descriptive data and statistical analysis obtained from male and female subjects with acute morbillous pancreatitis.

	Acute Morbillous Pancreatitis		*p*-Value
	Male (*n* = 14)	Female (*n* = 15)	
*Demographic characteristics*			
Mean age ± SD	37.4 ± 9.2	33.8 ± 10.1	>0.05
*Disease duration (mean days ±* *SD)*			
Total disease duration (days)	10.5 ± 3.9	13 ± 1.4	*0.038*
Hospitalization time (days)	8.1 ± 2.5	7.2 ± 2.4	>0.05
*Blood cell analyses (mean values ±* *SD)*			
Maximum WBC count (× 10^5^)	5.9 ± 2.5	6.2 ± 3.4	>0.05
Neutrophile percent abundance	78.2 ± 11.2	75.9 ± 15.4	>0.05
RBC count (× 10^12^)	4.7 ± 0.1	4.4 ± 0.5	*0.0053*
Hgb (g/L)	142.9 ± 4.2	131.1 ± 14.7	*0.003*
Platelets count (× 10^6^)	179 ± 97	164 ± 78	>0.05
*Serum biochemical analyses (mean values ±* *SD)*			
LDH (U/L)	903 ± 188	797 ± 203	>0.05
AST (U/L)	117 ± 54	105 ± 47	>0.05
ALT (U/L)	196 ± 25	130 ± 18	*<0.001*
γ-GT (U/L)	246 ± 31	240 ± 25	>0.05
Total bilirubin (μmol/L)	14.9 ± 5	8 ± 2.2	*<0.001*
Direct bilirubin (μmol/L)	6.2 ± 2.4	2.5 ± 0.2	*<0.001*
CRP before hospitalization (mg/L)	18.6 ± 9.1	45± 21	*<0.001*
CRP during hospitalization (mg/L)	42 ± 17	67.7 ± 23	*0.021*
Amylase (U/L)	386 ± 60	437 ± 76	*0.018*
*Urine biochemical analyses (mean values ±* *SD)*			
Amylase (U/L)	3450 ± 175	3200 ± 251	*0.0041*
*General measles-associated symptoms (number of cases (%))*			
Nausea (*n* (%))	13 (92.9)	11 (78.6)	>0.05
Vomiting (*n* (%))	12 (85.7)	10 (66.7)	>0.05
Diarrhea (*n* (%))	4 (28.5)	8 (52.8)	>0.05
Abdominal pain (*n* (%))	3 (21.4)	6 (39.6)	>0.05

WBC—white blood cells; RBC—red blood cells; Hgb—hemoglobin.

**Table 3:** Associations between CRP changes and the presence of acute morbillous pancreatitis.

		Acute Morbillous Pancreatitis		*p*-Value
	Category	Yes	No	
Changes in CRP	1	0%	3.1%	>0.05
	2	66.7%	55.4%	
	3	11.1%	13.8%	
	4	22.2%	27.7%	

CRP serum values increase: Category 1—less than 2-fold; Category 2—between 2- and 3-fold; Category 3—between 3- and 4-fold; Category 4—equal or larger than 4-fold.

## Data Availability

Data are available upon request.
